# Predictors of dropping out from a home tele-exercise programme: A cohort study derived from a randomised controlled trial

**DOI:** 10.34172/hpp.42935

**Published:** 2024-10-31

**Authors:** Gustavo Yuki, Luiz Hespanhol, Lisa Mohr, Adelle Kemlall Bhundoo, David Jiménez-Pavón, Bernhard Novak, Stefano Nuccio, Jose Daniel Jiménez García, Julian David Pillay, Lorenzo Rum, Celso Sánchez Ramírez, Lutz Vogt, Jan Wilke

**Affiliations:** ^1^Masters and Doctoral Programs in Physical Therapy, Universidade Cidade de São Paulo (UNICID), Sao Paulo, Brazil; ^2^Department of Physical Therapy, Faculty of Medicine, University of Sao Paulo (USP), Sao Paulo, Brazil; ^3^Amsterdam Collaboration on Health & Safety in Sports, Department of Public and Occupational Health, Amsterdam, Movement Sciences, Amsterdam University Medical Centers (UMC) location VU University Medical Center Amsterdam (VUmc), Amsterdam, The Netherlands; ^4^Department of Sports Medicine and Exercise Physiology, Goethe University Frankfurt, Frankfurt/Main, Germany; ^5^Department of Basic Medical Sciences, Durban University of Technology, South Africa; ^6^ImFine Research Group, Department of Health and Human Performance, Universidad Politécnica de Madrid; Exercise is Medicine, Spain; ^7^MOVE-IT Research Group, Department of Physical Education, Faculty of Education Sciences University of Cádiz, Spain; ^8^CIBER of Frailty and Healthy Aging (CIBERFES), Madrid, Spain; ^9^Institute of Human Movement Science, Sport and Health, University of Graz, Austria; ^10^Department of Movement, Human and Health Sciences, University of Rome “Foro Italico”, Rome, Italy; ^11^Sciences of Physical Activity, Sports and Health School, University of Santiago of Chile (USACH), Chile; ^12^Institute of Occupational, Social and Environmental Medicine, Goethe University Frankfurt, Frankfurt/Main, Germany

**Keywords:** Exercise, Health plan implementation, Implementation science, Social isolation, Telehealth

## Abstract

**Background:**

Online home exercises represent opportunities to increase physical activity levels. However, high dropout rates are commonly reported in such programmes. This study aimed to investigate the predictors of dropping out from an online home exercise programme.

**Methods:**

A total of 760 individuals from nine countries participated in this 8-week prospective cohort study derived from a randomised controlled trial. The participants were randomised into "4-week live-streamed exercise –>4-week recorded exercise" or "4-week no intervention –>4-week recorded exercise" group. Repeated measurements using weekly questionnaires were performed. Pain intensity, disability, mental well-being score, exercise motivation, sleep quality, impulsiveness/anxiety, and physical activity level were analysed.

**Results:**

A total of 53.8% (95% confidence interval [CI] 50.3%–57.3%) participants dropped out from the programme. The identified predictors of dropping out from the programme were: well-being (odds ratio [OR] 0.94, 95% CI 0.91–0.97) and disability (OR 1.02, 95% CI 1.002–1.04) at baseline considering the first 4 weeks; age (0.98; 95% CI 0.96–1.00) and baseline well-being (0.93; 95% CI 0.89–0.97) considering the entire follow-up (8 weeks); exercise motivation (0.92; 95% CI 0.87 to 0.97) and general impulsiveness/anxiety (1.04; 95% CI 1.01–1.07) repeated measured over time.

**Conclusion:**

About half of the participants dropped out from the online home exercise programme. Higher baseline scores in mental well-being and age predicted a reduction in dropping out. Higher baseline disability predicted an increase in dropping out. During the follow-up, higher exercise motivation was associated with a reduction in dropping out, and higher impulsiveness and anxiety were associated with an increase in dropping out.

## Introduction

 The increased use of Internet-enabled devices and their associated technologies has encouraged a wide spectrum of new opportunities for physical activity promotion.^1^ ‘eHealth’ seems to provide tools with the potential to reach a large number of people and it has been proven effective in increasing physical activity levels in young adults.^2^ Consequently, online home exercise represents an opportunity to be physically active. For example, 23% of a sample composed of 1508 Germans used digital sports activity programmes during the COVID-19 pandemic.^3^ The interest in participating in digital exercise programmes seems to be substantial, since a multicentre study found that 68.4% of the participants reported being interested in engaging in digital exercise programmes.^4^

 Regular physical activity practice promotes benefits in several health outcomes, such as hypertension, type 2 diabetes, mental health and sleep quality.^5^ Meeting physical activity guidelines is associated with a reduced risk of hospitalisation, intensive care unit admission and mortality.^6-8^ Conversely, declining levels of physical activity may increase the risk of comorbidities, such as cardiovascular diseases, respiratory diseases, and endocrine disorders.^9,10^

 Longitudinal studies are important in health research; however, high dropout rates are common in online home exercise programmes.^11-13^ Identifying the factors related to dropping out is essential for future implementation, considering that adherence may be an important mediator of the effects of exercise programmes on health outcomes. Therefore, this study aimed to investigate the predictors of dropping out from an online exercise intervention designed to increase or maintain physical activity levels.

## Methods

###  Study design

 This was a prospective cohort study derived from the ‘ ***A**ctivity and health during the **SA**rs-CoV2 **P**andemic*’ (ASAP) randomised controlled trial.^11^ The ASAP trial (move-ASAP) has been prospectively registered, and the protocol for the ASAP project can be found elsewhere.^14^ The data of the ASAP trial was treated in this study as an 8-week prospective cohort. This study was conducted in an international and multicentre setting, and the data were collected during 2020 in all nine countries enrolled (Argentina, Austria, Brazil, Chile, Germany, Italy, Ireland, South Africa, and Spain). The study was conducted according to the Declaration of Helsinki.^15^

###  Participants

 Participants were eligible for the study if: (1) they were ≥ 18 years; (2) they were from countries with officially registered cases of the novel coronavirus (SARS-CoV-2); (3) they were exposed to some form of social restriction; (4) they did not present any contraindication to unsupervised physical activity (e.g., severe orthopaedic, neurological, cardiovascular, metabolic, endocrine or psychiatric diseases^16^; or professional recommendation on engaging in physical activity only under medical supervision). The recruitment was performed on online social media (e.g., YouTube, Facebook, Instagram, WhatsApp, Telegram), mailing lists, and through dissemination and press releases from the universities and institutions of each centre/country involved.

###  Online exercise programme

 The online exercise programme was composed of different types of exercises, such as: flexibility, resistance, strength, mobility, coordination, and relaxation.^11^ The programme was based on the online exercise preferences indicated by participants from the target population, including the type of exercise, frequency, and duration of the workout sessions.^4^

###  Live-Streamed – > Recorded Exercise group (LSRE)

 The LSRE intervention was composed of two phases. During phase 1 (first 4-week period), all workouts were delivered to groups of participants through live-streaming sessions (synchronous mode) using a streaming software (e.g., Zoom, Microsoft Teams, Jitsi, or Blackboard). The participants could engage in workout sessions as much as they wished. However, the workout sessions were offered for a minimum of five days per week in each country (i.e., study weeks 1 to 4). Workouts were performed in groups, and they exhibited a multicomponent characteristic, including the following objectives: strength; endurance; flexibility; stability; balance; relaxation; and cognition. The duration of each multimodal workout session was approximately 45 minutes. During phase 2 (last 4-week period), all workouts were delivered to participants through *a priori* recorded sessions (asynchronous mode) using digital and online platforms (e.g., YouTube or university websites), and they were accessible 24 hours per day during the 4-week period. The participants could partake in any scheduled workout session, even more than once a day if they wished (i.e., study weeks 5 to 8).

###  No Intervention – > Recorded Exercise group (NIRE)

 The NIRE intervention was composed of two phases: (1) this group received no intervention during the first four weeks of the study (i.e., study weeks 1 to 4), when the participants were advised to wait these four weeks to receive the exercise programme; (2) the participants received the same intervention and mode as described in phase 2 of the LSRE intervention (i.e., study weeks 5 to 8).

###  Data collection and randomisation procedures

 At the beginning of the study, the participants were invited to complete an online baseline questionnaire. The randomisation and allocation into LSRE or NIRE group was performed automatically upon completing the baseline questionnaire, using a software algorithm embedded in the study online database (Soscisurvey, Soscisurvey GmbH, Munich, Germany). The follow-up online questionnaires were administered weekly throughout the study period (i.e., eight weeks).

###  Baseline and follow-up questionnaires

 Personal information such as age, sex, working place, living environment, university degree, and country of residence were collected only at baseline. The following instruments were used for measuring the specific outcome measures: the Chronic Graded Pain Scale (CGPS) for pain intensity and disability^17^; the World Health Organization – Five Well-Being Index (WHO-5) for mental well-being^18^; the self-concordance scale (SKK) for exercise motivation^19^; the Medical Outcomes Study 12-item Sleep Scale (MOS-12) for sleep quality^20^; the generalised anxiety disorder scale-7 (GAD-7) for impulsiveness and anxiety^21^; and the Nordic Physical Activity Questionnaire-short (NPAQ-short) for measuring the duration (minutes) spent performing moderate and vigorous physical activity.^22^

###  Dropout definition

 Dropout was defined as participants who withdrew from the study, regardless of the reason, either by actively declaring their wish to withdraw from the study, or by stopping answering, or even by never answering the follow-up questionnaires. If a participant did not answer the follow-up questionnaire, she/he was considered a dropout and no further invitations for the remaining weekly follow-up questionnaires ensued. That is, in cases where participants answered some follow-up questionnaires but stopped answering and did not return to answer in future follow-up questionnaires after two reminders (1 and 2 days after the correct date), they were considered dropouts from the time-point they stopped answering.

###  Data analysis

 Descriptive analyses were performed to summarise the baseline and follow-up data. Distributions were assessed by inspecting histograms and probability density functions. Continuous variables presenting an approximate normal distribution were summarised using means and the standard deviations (SD). Data presenting non-parametric distributions were summarised using medians and 25% to 75% interquartile range (IQR). Dichotomous and categorical variables were summarised using frequency distribution (n) and percentages (%).

 Logistic generalised models were performed to investigate the association between the baseline features and dropouts, and between the follow-up features and dropouts. We performed two logistic regression models to investigate the baseline predictors for dropping out during the first (weeks 1 to 4) or during the first and second (weeks 1 to 8) phases of the online exercise programme implementation. We performed logistic mixed models to investigate the longitudinal (time-dependent repeated measurements) association between the follow-up predictors and dropouts from the online exercise programme. All models included a ‘dropout’ indicator variable as the dependent variable. The mixed models (follow-up) included an indication variable for the repeated measurements in the random effects part of the model, and a time-lag technique was applied to allow the predictor of a given week predicting the dropout in the next week to guarantee that the predictor measurement came first to the outcome (dropout) measurement. All analyses were performed in R 3.5.0.^23^

## Results

**Figure 1:**
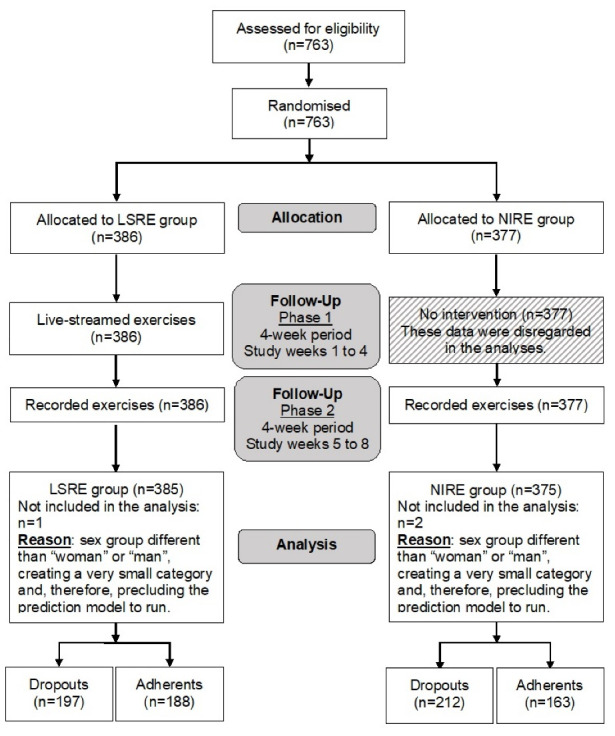
Flow of the participants during the study. LSRE, Live-Streamed –> Recorded Exercise; NIRE, No Intervention –> Recorded Exercise.

 The flow of the participants can be appreciated in Figure 1. A total of 760 participants were included in the analyses, 385 participants (50.7%) in the LSRE group and 375 participants (49.3%) in the NIRE group. A total of 409 participants (53.8%; 95% CI 50.3 to 57.3) dropped out from the online exercise programme. Personal and clinical data of the participants at baseline are described in Table 1. The participants of the study were mainly from Chile (30.0%, n = 228), Brazil (23.3%, n = 177) and Germany (16.4%, n = 125). The sample was mainly composed of women (68.8%, n = 523) and individuals working remotely (45.7%, n = 347). The median moderate physical activity level was 90 minutes per week (IQR 0 to 240).

**Table 1:** Characteristics of the participants at baseline

**Characteristics**	**Entire cohort (n=760)**	**LSRE group (n=385)**	**NIRE group (n=375)**
Age (years), mean (SD)	32.7 (12.6)	32.9 (13.1)	32.5 (12.0)
Sex, % (n)			
Woman	68.8% (523)	70.1% (270)	67.5% (253)
Man	31.2% (237)	29.9% (115)	32.5% (122)
Working place, % (n)			
Regular working place	10.0% (76)	9.4% (36)	10.7% (40)
Home-office	45.7% (347)	44.9% (173)	46.4% (174)
Mixed (regular and home-office)	30.7% (233)	29.4% (113)	32.0% (120)
Do not have a formal employment	12.0% (91)	14.0% (54)	9.9% (37)
Unwilling to disclose	1.7% (13)	2.3% (9)	1.1 (4)
Living environment, n (%)			
Rural	14.5% (110)	14.5% (56)	14.4% (54)
Urban	85.5% (650)	85.5% (329)	85.6% (321)
University degree, % (n)			
Yes	59.6% (453)	59.0% (227)	60.3% (226)
No	40.4% (307)	41.0% (158)	39.7% (149)
Pain intensity score (0–100), median (IQR)	10.0 (0.0 to 26.7)	13.3 (3.3 to 30.0)	10 (0.0 to 26.7)
Disability score (0–100), median (IQR)	0.0 (0.0 to 10.0)	0.0 (0.0 to 10.0)	0.0 (0.0 to 10.0)
Moderate physical activity (min), median (IQR)	90.0 (0.0 to 240.0)	90.0 (0.0 to 240.0)	90.0 (0.0 to 232.5)
Vigorous physical activity (min), median (IQR)	0.0 (0.0 to 82.5)	0.0 (0.0 to 90.0)	0.0 (0.0 to 75.0)
WHO-5 score (0–25), median (IQR)	13.0 (9.0 to 17.0)	13.0 (9.0 to 17.0)	13.0 (9.0 to 17.0)
Dropout, % (n)			
Yes	53.8% (409)	51.2% (197)	56.5% (212)
No	46.2% (351)	48.8% (188)	43.5% (163)
Country, % (n)			
Argentina	5.7% (43)	6.0% (23)	5.3% (20)
Austria	2.6% (20)	2.6% (10)	2.7% (10)
Brazil	23.3% (177)	23.1% (89)	23.5% (88)
Chile	30.0% (228)	29.6 (114)	30.4% (114)
Germany	16.4% (125)	16.4% (63)	16.5% (62)
Ireland	5.9% (45)	6.8% (26)	5.1% (19)
Italy	3.0% (23)	2.9% (11)	3.2% (12)
South Africa	9.6% (73)	9.4% (36)	9.9% (37)
Spain	3.4% (26)	3.4% (13)	3.5% (13)

LSRE, Live-Streamed – Recorded Exercise; NIRE, No Intervention – Recorded Exercise; WHO, World Health Organization – Five Well-Being Index (WHO-5); SD, standard deviation; IQR, 25% to 75% interquartile range.

Table 2 presents the results of the variables repeatedly measured during the follow-up. The pain intensity score was higher for the LSRE group than for the NIRE group at week 1 and 8. Regarding the physical activity levels, the LSRE group was more active compared to the NIRE group at any time-point during the study. During phase 2 of the study, WHO-5 scores were lower compared to phase 1.

 The results of the logistic mixed models can be found in Table 3. Regarding baseline predictors, WHO-5 score (0.94, 95% CI 0.91 to 0.97) and disability score (1.02, 95% CI 1.002 to 1.04) at baseline were associated with dropping out from the online exercise programme during the phase 1 (first 4-week period). Considering the entire follow-up period (i.e., 8 weeks), baseline values for WHO-5 score (0.93, 95% CI 0.89 to 0.97) and age (0.98, 95% CI 0.96 to 1.00) were associated with dropping out from the online exercise programme. Regarding follow-up predictors, SKK (0.92, 95% CI 0.87 to 0.97) and GAD-7 (1.04, 95% CI 1.01 to 1.07) were associated with dropping out from the online exercise programme. Participants from Brazil and Austria presented the lowest odds of dropping out from the online exercise programme at any given time-point (Table 3).

**Table 2:** Variables repeatedly measured over the follow-up

**Variables**	**Phase 1**				**Phase 2**			
	**Week 1**	**Week 2**	**Week 3**	**Week 4**	**Week 5**	**Week 6**	**Week 7**	**Week 8**
Pain intensity score (0–100), median (IQR)								
Entire cohort	13.3 (0.0 to 33.3) n = 478	10.0 (0.0 to 30) n = 428	10.0 (10.0 to 26.7) n = 377	6.7 (0.0 to 23.3) n = 349	6.7 (0.0 to 23.3) n = 303	6.7 (0.0 to 23.3) n = 267	6.7 (0.0 to 20.0) n = 245	6.7 (0.0 to 20.0) n = 228
LSRE group	20.0 (6.7 to 36.7) n = 249	13.3 (0.0 to 30) n = 221	10.0 (0.0 to 26.7) n = 201	6.7 (0.0 to 23.3) n = 189	6.7 (0.0 to 30) n = 158	6.7 (0.0 to 23.3) n = 141	6.7 (0.0 to 20) n = 129	8.3 (0.0 to 20) n = 118
NIRE group	10.0 (0.0 to 30.0) n = 229	10.0 (0.0 to 26.7) n = 207	10.0 (0.0 to 26.7) n = 176	6.7 (0.0 to 23.3) n = 160	6.7 (0.0 to 20.0) n = 145	8.3 (0.0 to 22.5) n = 126	6.7 (0.0 to 20.0) n = 116	3.3 (0.0 to 16.7) n = 110
Disability score (0–100), %; n								
Entire cohort	n = 478	n = 428	n = 377	n = 349	n = 303	n = 267	n = 245	n = 228
0–5	61.5%; n = 294	70.8%; n = 303	72.9%; n = 275	75.4%; n = 263	75.2%; n = 228	78.7%; n = 210	75.9%; n = 186	78.5%; n = 179
6–10	15.5%; n = 74	10.5%; n = 45	9%; n = 34	9.7%; n = 34	8.9%; n = 27	7.5%; n = 20	8.6%; n = 21	9.2%; n = 21
11–25	11.9%; n = 57	7.9%; n = 34	8%; n = 30	7.7%; n = 27	6.3%; n = 19	5.6%; n = 15	7.3%; n = 18	8.8%; n = 20
26–50	7.7%; n = 37	7.9%; n = 34	8.5%; n = 32	5.2%; n = 18	6.3%; n = 19	6%; n = 16	5.7%; n = 14	2.6%; n = 6
51–75	2.7%; n = 13	2.1%; n = 9	1.1%; n = 4	1.4%; n = 5	2.6%; n = 8	1.5%; n = 4	1.2%; n = 3	0.9%; n = 2
76–100	0.6%; n = 3	0.7%; n = 3	0.5%; n = 2	0.6%; n = 2	0.7%; n = 2	0.7%; n = 2	1.2%; n = 3	0.0%; n = 0
LSRE group	n = 249	n = 221	n = 201	n = 189	n = 158	n = 141	n = 129	n = 118
0–5	60.2%; n = 150	68.3%; n = 151	69.2%; n = 139	74.1%; n = 140	73.4%; n = 116	80.1%; n = 113	75.2%; n = 97	77.1%; n = 91
6–10	15.7%; n = 39	10.4%; n = 23	10.9%; n = 22	12.2%; n = 23	9.5%; n = 15	6.4%; n = 9	9.3%; n = 12	10.2%; n = 12
11–25	12%; n = 30	8.6%; n = 19	7.5%; n = 15	7.9%; n = 15	7%; n = 11	5.7%; n = 8	7%; n = 9	9.3%; n = 11
26–50	9.2%; n = 23	10%; n = 22	10.0%; n = 20	2.6%; n = 5	6.3%; n = 10	5.7%; n = 8	6.2%; n = 8	2.5%; n = 3
51–75	2.4%; n = 6	1.8%; n = 4	1.5%; n = 3	2.1%; n = 4	2.5%; n = 4	0.7%; n = 1	0.0%; n = 0	0.8%; n = 1
76–100	0.4%; n = 1	0.9%; n = 2	1.0%; n = 2	1.1%; n = 2	1.3%; n = 2	1.4%; n = 2	2.3%; n = 3	0.0%; n = 0
NIRE group	n = 229	n = 207	n = 176	n = 160	n = 145	n = 126	n = 116	n = 110
0–5	62.9%; n = 144	73.4%; n = 152	77.3%; n = 136	76.9%; n = 123	77.2%; n = 112	77%; n = 97	76.7%; n = 89	80%; n = 88
6–10	15.3%; n = 35	10.6%; n = 22	6.8%; n = 12	6.9%; n = 11	8.3%; n = 12	8.7%; n = 11	7.8%; n = 9	8.2%; n = 9
11–25	11.8%; n = 27	7.2%; n = 15	8.5%; n = 15	7.5%; n = 12	5.5%; n = 8	5.6%; n = 7	7.8%; n = 9	8.2%; n = 9
26–50	6.1%; n = 14	5.8%; n = 12	6.8%; n = 12	8.1%; n = 13	6.2%; n = 9	6.3%; n = 8	5.2%; n = 6	2.7%; n = 3
51–75	3.1%; n = 7	2.4%; n = 5	0.6%; n = 1	0.6%; n = 1	2.8%; n = 4	2.4%; n = 3	2.6%; n = 3	0.9%; n = 1
76–100	0.9%; n = 2	0.5%; n = 1	0.0%; n = 0	0.0%; n = 0	0.0%; n = 0	0.0%; n = 0	0.0%; n = 0	0.0%; n = 0
Moderate physical activity (min), median (IQR)								
Entire cohort	140.0 (25.0 to 360.0) n = 476	50.0 (0.0 to 240.0) n = 425	150.0 (30.0 to 300.0) n = 373	150.0 (37.7 to 300.0) n = 342	150.0 (45.0 to 310.0) n = 303	180.0 (60.0 to 350.0) n = 265	160.0 (50.0 to 350.0) n = 243	180.0 (47.5 to 360.0) n = 227
LSRE group	200.0 (60.0 to 400.0) n = 249	95.0 (0.0 to 300.0) n = 220	180.0 (60.0 to 375.0) n = 197	180.0 (60.0 to 347.5) n = 187	190.0 (60.0 to 395.0) n = 158	200.0 (80.0 to 400.0) n = 140	200.0 (60.0 to 380.0) n = 129	200.0 (60.0 to 390.0) n = 118
NIRE group	75.0 (0.0 to 245.0) n = 227	0.0 (0.0 to 165.0) n = 205	115.0 (0.0 to 240.0) n = 176	120.0 (0.0 to 245.0) n = 155	120.0 (20.0 to 240.0) n = 145	120.0 (30.0 to 270.0) n = 125	125.0 (40.0 to 240.0) n = 114	120.0 (30.0 to 250.0) n = 109
Vigorous physical activity (min), median (IQR)								
Entire cohort	30.0 (0.0 to 120.0) n = 476	0.0 (0.0 to 70.0) n = 425	30.0 (0.0 to 120.0) n = 373	30.0 (0.0 to 147.5) n = 342	30.0 (0.0 to 132.5) n = 303	45.0 (0.0 to 130.0) n = 265	40.0 (0.0 to 150.0) n = 243	40.0 (0.0 to 150.0) n = 227
LSRE group	50.0 (0.0 to 165.0) n = 249	(0.0 to 120.0) n = 220	60.0 (0.0 to 160.0) n = 197	60.0 (0.0 to 160.0) n = 187	55.0 (0.0 to 180.0) n = 158	60.0 (7.5 to 200.0) n = 140	50.0 (0.0 to 200.0) n = 129	60.0 (0.0 to 200.0) n = 118
NIRE group	0.0 (0.0 to 60.0) n = 227	0.0 (0.0 to 50.0) n = 205	10.0 (0.0 to 60.0) n = 176	20.0 (0.0 to 110.0) n = 155	20.0 (0.0 to 90.0) n = 145	20.0 (0.0 to 90.0) n = 125	30.0 (0.0 to 120.0) n = 114	20.0 (0.0 to 120.0) n = 109
WHO-5 score (0–25), median (IQR)								
Entire cohort	15.0 (10.0 to 19.0) n = 472	15.0 (11.0 to 19.0) n = 424	16.0 (11.0 to 19.0) n = 373	17.0 (12.0 to 20.0) n = 342	13.0 (10.0 to 18.0) n = 300	13.0 (10.0 to 17.0) n = 265	12.0 (10.0 to 17.0) n = 244	12.0 (10.0 to 16.2) n = 228
LSRE group	15.0 (10.0 to 19.0) n = 246	15.0 (11.0 to 20.0) n = 219	16.0 (11.0 to 20.0) n = 198	17.0 (12.0 to 20.0) n = 186	13.0 (10.0 to 17.0) n = 156	13.0 (10.0 to 16.2) n = 140	12.0(10.0 to 16.0) n = 129	11.5 (10.0 to 16.0) n = 120
NIRE group	15.0 (10.0 to 19.0) n = 226	15.0 (11.0 to 19.0) n = 205	15.0 (11.0 to 19.0) n = 175	16.0 (11.0 to 20.0) n = 156	14.0 (10.0 to 19.0) n = 144	13.0 (10.0 to 17.0) n = 125	13.0 (10.0 to 17.0) n = 115	12.5 (10.0 to 17.2) n = 108
SKK- Index (-10–10), mean (SD)								
Entire cohort	4.4 (2.6) n = 477	5.7 (5.2) n = 427	4.4 (3.0) n = 376	4.5 (2.8) n = 347	4.4 (2.8) n = 303	4.3 (3.0) n = 267	4.4 (2.8) n = 245	4.4 (3.0) n = 228
LSRE group	4.3 (2.6) n = 249	5.9 (5.5) n = 220	4.4 (3.1) n = 200	4.5 (2.8) n = 188	4.6 (2.8) n = 158	4.4 (3.1) n = 141	4.5 (2.8) n = 129	4.7 (2.7) n = 118
NIRE group	4.5 (2.6) n = 228	5.5 (4.9) n = 207	4.3 (2.8) n = 176	4.4 (2.9) n = 159	4.2 (2.8) n = 145	4.1 (2.9) n = 126	4.3 (3.0) n = 116	4.1 (3.3) n = 110
MOS sleep scale II (0–100), mean (SD)								
Entire cohort	67.6 (12.1) n = 477	68.8 (10.7) n = 428	68.1 (11.5) n = 377	68.7 (10.1) n = 349	67.9 (11.3) n = 303	66.6 (12.5) n = 267	69.0 (10.6) n = 245	69.0 (11.2) n = 228
LSRE group	66.8 (12.0) n = 248	69.3 (10.4) n = 221	68.1 (11.1) n = 201	68.7 (10.2) n = 189	67.4 (12.6) n = 158	66.1 (13.1) n = 141	68.9 (11.4) n = 129	70.0 (11.2) n = 118
NIRE group	68.5 (12.2) n = 229	68.3 (11.0) n = 207	68.1 (11.9) n = 176	68.7 (10.2) n = 160	68.4 (9.9) n = 145	67.1 (11.8) n = 126	69.1 (9.7) n = 116	68.0 (11.1) n = 110
GAD 7 questionnaire (0–21), median (IQR)								
Entire cohort	6.0 (3.0 to 9.0) n = 478	5.0 (2.0 to 9.0) n = 428	4.0 (2.0 to 8.0) n = 377	4.0 (1.0 to 8.0) n = 349	4.0 (2.0 to 7.0) n = 303	4.0 (1.0 to 7.0) n = 267	3.0 (1.0 to 7.0) n = 245	4.0 (1.0 to 7.0) n = 228
LSRE group	5.0 (2.0 to 9.0) n = 249	4.0 (2.0 to 8.0) n = 221	4.0 (2.0 to 8.0) n = 201	4.0 (1.0 to 8.0) n = 189	4.0 (1.0 to 7.0) n = 158	3.0 (1.0 to 7.0) n = 141	3.0 (1.0 to 7.0) n = 129	3.0 (0.0 to 7.0) n = 118
NIRE group	6.0 (3.0 to 9.0) n = 229	6.0 (3.0 to 9.0) n = 207	5.0 (2.0 to 7.2) n = 176	4.0 (2.0 to 7.0) n = 160	4.0 (2.0 to 6.0) n = 145	4.0 (1.0 to 7.0) n = 126	3.0 (1.0 to 6.0) n = 116	4.0 (2.0 to 7.0) n = 110

GAD 7, Generalized Anxiety Disorder scale-7; MOS, Medical Outcomes Study 12-item Sleep Scale; SKK, Self-concordance Scale for Exercise Motivation; WHO, World Health Organization – Five Well-Being Index (WHO-5); LSRE, Live-Streamed – > Recorded Exercise; NIRE, No Intervention – > Recorded Exercise; IQR, 25% to 75% interquartile range.

**Table 3:** Mixed models results on the prediction of dropouts from the online exercise intervention

**Variables**	**Baseline predictors**		**Follow-up predictors**
	**4 weeks** **OR (95% CI)**	**8 weeks** **OR (95% CI)**	**Mixed models** **OR (95% CI)**
Intercept	7.77 (2.04 to 29.64)	32.60 (6.05 to 175.85)	1.24 (0.28 to 5.45)
Sex	0.85 (0.57 to 1.27)	1.18 (0.77 to 1.83)	–
Age (years)	0.99 (0.98 to 1.01)	0.98 (0.96 to 1.00)*	–
Working place	1.06 (0.92 to 1.23)	0.97 (0.82 to 1.15)	–
Living environment	0.79 (0.48 to 1.29)	0.87 (0.49 to 1.57)	–
University degree	0.79 (0.55 to 1.15)	1.26 (0.83 to 1.92)	–
Pain intensity score (0–100)	0.99 (0.98 to 1.00)	1.00 (0.98 to 1.01)	0.99 (0.98 to 1.01)
Disability score (0–100)	1.02 (1.002 to 1.04)*	1.02 (0.99 to 1.04)	1.01 (0.997 to 1.02)
Moderate physical activity (min)	1.000 (0.999 to 1.001)	0.999 (0.998 to 1.001)	0.999 (0.998 to 1.000)
Vigorous physical activity (min)	1.001 (1.000 to 1.003)	1.001 (0.999 to 1.003)	1.002 (1.000 to 1.003)
WHO-5 score (0–25)	0.94 (0.91 to 0.97)*	0.93 (0.89 to 0.97)*	1.01 (0.98 to 1.04)
SKK- Index (-10–10)	–	–	0.92 (0.87 to 0.97)*
MOS sleep scale II (0–100)	–	–	1.00 (0.99 to 1.01)
GAD 7 questionnaire (0–21)	–	–	1.04 (1.01 to 1.07)*
Delivery mode			
Synchronous	–	–	Reference
Asynchronous	–	–	0.94 (0.64 to 1.38)
Country			
Italy	Reference	Reference	Reference
Argentina	1.26 (0.39 to 4.06)	1.39 (0.28 to 6.89)	1.04 (0.32 to 3.39)
Austria	0.18 (0.04 to 0.72)*	0.09 (0.02 to 0.45)*	0.14 (0.03 to 0.55)*
Brazil	0.09 (0.03 to 0.25)*	0.05 (0.01 to 0.19)*	0.06 (0.02 to 0.17)*
Chile	1.99 (0.73 to 5.43)	1.47 (0.38 to 5.69)	1.36 (0.51 to 3.69)
Germany	0.44 (0.16 to 1.20)	0.28 (0.08 to 1.05)	0.30 (0.11 to 0.83)*
Ireland	0.52 (0.16 to 1.62)	1.05 (0.21 to 5.27)	0.59 (0.19 to 1.87)
South Africa	1.18 (0.40 to 3.47)	0.84 (0.20 to 3.50)	0.60 (0.20 to 1.77)
Spain	0.15 (0.04 to 0.56)*	0.48 (0.10 to 2.31)	0.34 (0.10 to 1.13)

CI, Confidence interval; ‘–*’*, Variables not included in the model because they were only measured at baseline or during the follow-up. ‘*’, Statistically significant.

## Discussion

 This study aimed at investigating the predictors of dropping out from an online home exercise programme designed to increase or maintain physical activity levels. The proportion of dropping out from the online home exercise programme was 53.8% (95% CI 50.3 to 57.3). The LSRE group presented higher levels of physical activity compared to NIRE group during the entire follow-up. Higher mental well-being scores and higher age at baseline were predictors for lower odds of dropping out from the online exercise programme: for each 1-point increase in mental well-being score (WHO-5), the dropout odds reduced by 7% (95% CI ≈3% to 12%); for each year increase in age, the dropout odds reduced by 2% (95% CI 0% to 4%). Also, a 1-point increase in disability score at baseline predicted higher dropout odds by 2% (95% CI 0% to 4%) after 4 weeks of online exercise implementation. During the follow-up, exercise motivation, impulsiveness and anxiety were predictors for dropping out from the online exercise programme: for each 1-point increase in exercise motivation (SKK), the dropout odds reduced by 8% (95% CI 3% to 13%); for each 1-point increase in impulsiveness and anxiety (GAD-7), the dropout odds increased by 4% (95% CI 1% to 7%).

 Online intervention programmes aimed to encourage physical activity practice have shown positive results, especially in the amount of physical activity (physical activity levels,^1,11,13,24-27^ number of steps,^1,26,27^ and minutes of walking^26^), quality of life,^24,25^ mental well-being score (WHO-5),^11^ anxiety (GAD-7),^11^ sleep quality (MOS-2),^11^ and exercise motivation (SKK).^11^ Randomised controlled trials are essential to evidence-based practice, but they commonly have high dropout rates, especially effectiveness trials mimicking real context for implementation.^28,29^ This was the case for the online exercise programme investigated by this project where the trial (efficacy) results were reported elsewhere.^11^ Dropouts should be considered when interpreting the results of randomised controlled trials, because these rates may compromise the internal and/or external validity of the results. Furthermore, dropout proportions for online interventions may vary depending on the target-population, such as pulmonary diseases (about 57% of dropout rate),^12^ musculoskeletal conditions (about 14% of dropout rate),^30^ and healthy subjects (about 22.5% of dropout rate).^13^ Enhancing features of physical activity programmes related to education information, self-monitoring, goal setting, commitment, and receiving feedback may influence behavioural changes to improve engagement and decrease dropouts.

 The delivery mode of the online exercise programme was not associated as a predictor for dropouts. This finding did not corroborate our *a priori* hypothesis. We initially hypothesised that synchronous exercise would be associated with lower odds for dropping out from the online exercise programme. Based on the results of this study, delivering online exercise programmes synchronously or asynchronously would not influence the dropout odds. However, the delivery mode period was rather short (i.e., 4 weeks for either synchronous or asynchronous mode), and maybe the 4-week period was not sufficient for eliciting dropping out differences between synchronous and asynchronous delivery modes. Nevertheless, there is evidence corroborating that there is no difference between remote and face-to-face exercise delivery modes regarding levels of physical activity during periods of social restriction.^31^

 Individuals with higher levels of depression and/or anxiety are usually those who report less self-motivation for engaging in physical activity.^32,33^ Our results corroborate this rationale, since the participants reporting higher mental well-being scores at baseline were those with lower odds for dropping out from the online home exercise programme. In consonance, those reporting higher exercise motivation and higher impulsiveness and anxiety during the follow-up were those with lower and higher odds for dropping out from the online exercise programme, respectively. The association between mental health and physical activity compliance seems to present a 2-way relationship, since it has been shown that reduced levels of physical activity were associated with a reduction in mental well-being scores.^34^ Therefore, lack of exercise motivation and symptoms of depression and/or anxiety may be major barriers to exercise engagement.

 Higher age seems to be a predictor for continuing to exercise.^35,36^ Our results indicated that a 1-year increase in age was associated with a reduction of dropping out from the online exercise programme by 2% (95% CI 0% to 4%) during the 8-week period. Based on this estimate, a 10-year increase in age would result in a reduction by 20% in dropping out from exercise programmes, effect size that corroborate with previous evidence.^35^ Some factors might help explaining this evidence^35^: (1) as time passes, individuals tend to manage time better; (2) usually they become more stable in their work and/or careers; and (3) they give more value to social interactions.

 Our results suggest that higher levels of disability at baseline may increase the odds of dropping out from online exercise programmes. People with higher levels of disability may present a higher perceived risk and/or higher fear of discomfort related to the participation in online exercise programmes. In patients with chronic low back pain, for example, higher disability rates at baseline has led to lower adherence to home exercises,^37^ corroborating our findings. Also, our results suggest that those who might benefit most from practising exercises were those who dropped out more often from our online exercise programme (i.e., lower exercise motivation, higher levels of disability, lower mental well-being scores, and higher impulsiveness and anxiety). This highlights the need to design and implement exercise programmes targeting specific groups aimed at reducing barriers and, therefore, facilitating exercise participation for those who would benefit the most.

 A strength of this study is that we conducted an international multicentre longitudinal study during an atypical period when the maintenance of physical activity levels was affected,^38-40^ and one way to help in overcoming this problem was to offer an online and home-based exercise programme to the population.^11^ The methods of the study allowed the participants to do the sessions at an undetermined frequency and at the best time in their routine, mimicking the ‘real world’ context. However, the study had some limitations. The follow-up period for eliciting health benefits related to the enrolment in the online exercise programme was short. On one hand, a possible lack of perceived health benefit could have been a reason for dropping out from the online exercise programme. On the other hand, long periods of follow-up could have led to a higher dropout rate due to, for example, lack of interest in maintaining a home and online exercise programme for a long time. The ‘burden’ associated with the weekly questionnaires may have discouraged some participants from continuing the online exercise programme.

## Conclusion

 ‘eHealth’ can provide tools with the potential to reach many people regarding physical activity and health promotion actions. However, high dropout rates are common in digital home exercise programmes, as evidenced by this study showing that about half of the participants dropped out from the online home exercise programme.

 Identifying the factors related to dropping out from exercise programmes is essential for the success of future implementation, considering that adherence may be an important mediator of the effects of exercise interventions on health outcomes. In this study, a 1-point increase in mental well-being score at baseline was associated with a reduction in dropping out from online exercise intervention by 6% in 4 weeks and by 7% in 8 weeks. A 1-point increase in disability score at baseline was associated with an increase in dropping out from online exercise intervention by 2% in 4 weeks. A 1-point increase in age at baseline was associated with a reduction in dropping out from online exercise intervention by 2% in 8 weeks. During the follow-up, a 1-point increase in exercise motivation was associated with a reduction in dropping out from online exercise intervention by 8% and a 1-point increase in impulsiveness and anxiety was associated with an increase in dropping out from online exercise intervention by 4%.

 Therefore, strategies for enhancing adherence to online and digital home exercise programmes, such as education, self-monitoring, goal setting, and providing feedback should be employed especially for the younger, those with lower mental well-being, lower exercise motivation, higher disability, and higher impulsiveness/anxiety.

## Acknowledgments

 Chiara Fossati (Italian study centre) and Mireille van Poppel (Austrian study centre) provided valuable administrative support. Furthermore, the authors thank: Lisandra Almeida, Rafael Olivera and Henrique Martins Ungri (Brazil); Patricia Sheehan, Annalouise Muldoon and Niamh Murphy (Ireland); Sonia Ortega-Gómez (Spain); David Url (Austria); Fernando Laiño (Argentina); Falk Richter, Marie Wilke and Leander Sielaff (Germany); for their assistance in conducting the exercise sessions.

## Competing Interests

 The authors declare that they have no conflict of interest of any nature regarding this study.

## Ethical Approval

 Ethics approval was obtained in each centre (Ethics Committee of the Faculty of Psychology and Sports Sciences of Goethe University Frankfurt, protocol no. 2020-25; Ethics Committee of Karl Franzens University Graz, no. 39/66/63 ex 2019/20; Comitato di Ateneo per la Ricerca, Università degli Studi di Roma “Foro Italico”, no. CAR 45/2020; Research Ethics Committee of the Universidade Cidade de São Paulo, no. 31216720.2.000.0064; Institutional Research Ethics Committee of Durban University of Technology, no. IREC 090/20; Institutional Ethics Committee University of Santiago of Chile, no. 207/2020; Research Ethics Committee of Fundación Instituto Superior de Ciencias de la Salud, no. DEPINV12/20; Research Ethics Committee Waterford Institute of Technology no WIT2020REC100; Ethics Committee of the Universidad Politécnica de Madrid).
